# Global North learning from Global South: A community-led response to mpox in New York City

**DOI:** 10.1371/journal.pgph.0002042

**Published:** 2023-06-28

**Authors:** Grant Roth, Jennifer Barnes-Balenciaga, Joseph Osmundson, Martez D. R. Smith, Nguyen K. Tran, Nicholas Diamond, Keletso Makofane

**Affiliations:** 1 RESPND-MI Study Team, New York, New York, United States of America; 2 Project ACHIEVE, New York, New York, United States of America; 3 Crystal La’Beija Organizing Fellowship, New York, New York, United States of America; 4 New York University, New York, New York, United States of America; 5 University of Rochester, Rochester, New York, United States of America; 6 The PRIDE Study/PRIDEnet, Stanford University, Stanford, California, United States of America; 7 Harvard University, Cambridge, Massachusetts, United States of America; PLOS: Public Library of Science, UNITED STATES

Global health practice is rooted in a colonial understanding and use of knowledge; organizations in the Global North produce knowledge for use in the Global South, whereas knowledge produced in the Global South is often overlooked in the Global North. Over the past decade, however, some of the authors of this piece have participated in a successful effort by key population civil society organizations to root global HIV program and policy guidance in the experiences of experts who practice in the Global South. We reflect on the influence of principles elaborated in this work implicitly shaped our community-led response to the global mpox epidemic as experienced in New York City.

## The Mpox outbreak and the government’s response

Mpox cases in the United States (U.S.) were first detected in May 2022 and rapidly escalated, with over 10,000 cases reported by the end of July 2022 [1]. Initial clusters were associated with large gatherings involving sex and concentrated in white, cisgender, gay, bisexual, and other men who have sex with men, with limited data available for minoritized ethno-racial and transgender communities. Over time, new data indicated that Black, Latinx, and transgender communities were also disproportionately affected [2].

Despite early warnings from activists about the lack of testing capacity in the U.S., responses from city, state, and federal governments were slow and inadequate [3]. At the height of Pride month in June, only about 20 mpox tests were administered daily in New York City (NYC), in stark contrast to the 719 probable or confirmed mpox cases in NYC between May 19 to July 15, 2022 [4]. This was a critical period for scaling prevention efforts in vulnerable communities affected by mpox, yet structural divestments led to inadequate data collection; unpredictable vaccination access and vaccine shortages [5]; and challenges in obtaining and providing treatment [6].

## How global health activism informed our community’s response

In response to these challenges, a group of queer and transgender NYC-based activists formed the Rapid Epidemiologic Study of Prevalence, Network, and Demographics of Mpox Infection (RESPND-MI). The goals of RESPND-MI were to 1) measure the outbreak’s demographics and spatial epidemiology; and 2) address gaps in mpox vaccine and treatment access due to flawed testing data and an outdated understanding of group sex among queer and transgender communities.

Our approach to preparing for the study was strongly influenced by principles and practices that have been elaborated in global health advocacy over the past decade and codified in several guidance documents. We highlight three principles here: community empowerment, community leadership, and accountability.

Community empowerment involves “increasing… communities’ control over their health by addressing the structural constraints to health, human rights, and wellbeing” [7]. Adopting a community empowerment framework entails promoting a sex-positive approach; facilitating affinity; leveraging the talents of community members; and trusting that they know how best to identify priorities and strategies to address the problems they face [8]. Belief in community leadership means recognizing that the power to make consequential decisions should be placed in the hands of those whose lives will be affected by those decisions [8]. It also involves fostering autonomy, allowing for ownership of the process, including decisions about the dissemination and use of the generated data [9]. Accountability involves implementing various collective strategies to hold actors in the healthcare system accountable and align their actions with their mandates.

## RESPND-MI study team and the community forum

We organized the RESPND-MI study team into three working groups, inviting community members to join regardless of academic credentials: the administration group (fundraising and Institutional Review Board applications); the technical group (questionnaire and survey instrument development); and the external affairs group (public-facing marketing, communications, and partnerships). Community partners joined as co-investigators and participated in all collective decision-making.

The external affairs group played a crucial role in ensuring that RESPND-MI benefited from the expertise of individuals and organizations working in queer and transgender health in NYC. The team regularly consulted with stakeholders, including activists, government officials, community-based organizations, clinicians, researchers, and other community partners through the RESPND-MI’s community forum.

Consulting with community partners in the forum structured RESPND-MI’s effort in three concrete ways. First, a Spanish version of the survey was developed since about a quarter of New York residents speak Spanish at home [10]. Second, a community partner, who later joined as a co-investigator, provided invaluable guidance on engaging with transgender individuals in all aspects of RESPND-MI. This marked a significant shift in the approach, which had previously only focused on cisgender gay and bisexual men. Third, feedback from the community forum informed the design of study materials, including the selection of a name and marketing concept that conveyed scientific rigor, avoided stigmatizing sexual practices, and had visual appeal. The collective input from the community forum greatly influenced the study’s approach and enhanced its relevance and sensitivity to community needs.

The community forum quickly evolved from a consultation to a coordination mechanism, as participants expressed the need for a regular space to receive mpox updates and share their experiences. Through ongoing discussions, collective needs related to the outbreak were identified, leading to collaborations to address those needs. The forum produced and disseminated valuable outputs, including a vaccination locator [11], safer-sex guidance [12], and a policy brief [13].

## Towards a shared knowledge

Our decision to initiate this project, rather than relying on government agencies to address the data gaps, was driven by the principle of community empowerment and leadership. These principles undergirded the decisions to amplify community-generated resources that were often timelier and more relevant than those released by public health agencies; respond to well-founded criticisms that respect the dignity of queer and transgender people; engage community members regardless of academic credentials as co-investigators rather than informants; and distribute responsibility for crucial aspects of the study among all investigators.

We fostered accountability by collecting data to ensure that public health agencies serve our communities well [9, 14]. We prioritized creating and sustaining the community forum for information exchange, coordination of collective action, and holding the government accountable. While the latter activity may appear peripheral to the study objectives, it reinforces our primary aim to influence how the government serves queer and transgender communities in the absence of sufficient messaging and interventions.

Amidst the ongoing examination of colonialism’s legacy in global health, our project serves as a testament to the longstanding efforts of activists to integrate radical ideas into the field. This work also highlights the successful application of knowledge developed by civil society organizations, particularly from the Global South, in the Global North. We are not aware of other projects in the U.S. that responded as quickly and as comprehensively to mpox, while being led by the community. While the field of global health largely operates under the assumption that the role of people of the Global North is to extend charity to those in the Global South, we highlight the potential to productively apply in the North, knowledge generated in the South. We do so in the hopes of highlighting that all people, in the north and south, stand to gain from efforts to decolonize global health.
